# Replicability and generalizability in population psychiatric neuroimaging

**DOI:** 10.1038/s41386-024-01960-w

**Published:** 2024-08-30

**Authors:** Scott Marek, Timothy O. Laumann

**Affiliations:** 1 Mallinckrodt Institute of Radiology, Washington University in St. Louis School of Medicine, St. Louis, MO USA; 2 Department of Psychiatry, Washington University in St. Louis School of Medicine, St. Louis, MO USA; 3 Neuroimaging Labs Research Center, Washington University in St. Louis School of Medicine, St. Louis, MO USA; 4 AI Institute for Health, Washington University in St. Louis School of Medicine, St. Louis, MO USA

**Keywords:** Cognitive neuroscience, Predictive markers

## Abstract

Studies linking mental health with brain function in cross-sectional population-based association studies have historically relied on small, underpowered samples. Given the small effect sizes typical of such brain-wide associations, studies require samples into the thousands to achieve the statistical power necessary for replicability. Here, we detail how small sample sizes have hampered replicability and provide sample size targets given established association strength benchmarks. Critically, while replicability will improve with larger samples, it is not guaranteed that observed effects will meaningfully apply to target populations of interest (i.e., be generalizable). We discuss important considerations related to generalizability in psychiatric neuroimaging and provide an example of generalizability failure due to “shortcut learning” in brain-based predictions of mental health phenotypes. Shortcut learning is a phenomenon whereby machine learning models learn an association between the brain and an unmeasured construct (the shortcut), rather than the intended target of mental health. Given the complex nature of brain-behavior interactions, the future of epidemiological approaches to brain-based studies of mental health will require large, diverse samples with comprehensive assessment.

## Introduction

A predominant strategy for investigating the neurobiology of mental health has been to associate brain features with phenotypic traits that may relate to psychopathology [1]. Studies using this population neuroscience framework to psychiatric neuroimaging most often report on brain-behavior associations or phenotype prediction [2]. Association studies link an aspect(s) of brain structure (e.g., volume, thickness) or function (e.g., resting-state functional connectivity (RSFC) and task fMRI) with a wide array of behavioral phenotypes, including cognition and mental health diagnoses or symptom profiles. Prediction/postdiction studies leverage machine learning to build multivariate brain-based models of psychopathology [3], usually at the individual difference level rather than the group level. Understanding patterns of association and participant-level prediction based in brain imaging may inform biomarker development and clinical translation [4].

Classically, population psychiatric neuroimaging studies have reported effects based on small samples (i.e., tens to a few hundred participants) [5, 6]. Our central thesis is that, for measuring population-level psychopathologic variability in brain function, small samples lack the replicability and generalizability required for ultimate clinical translation. In this perspective, we will explore the mechanisms underlying the link between small sample sizes and poor replicability in population studies. We will then provide empirical evidence for the effect size of the normative relationship between brain function (as measured by RSFC) and psychopathology to provide a sense of the sample sizes required for improved replicability. Further, we argue that large samples alone will not ensure generalizability, a high benchmark needed for eventual clinical utility. We conclude with paths forward for population psychiatric neuroimaging, noting distinctions from cohort, within-person and intervention designs.

## Mechanisms linking small sample sizes to replication failures

All scientific enterprises seek high replicability and external validity (hereafter referred to as generalizability). Replicability and generalizability are related, but distinct concepts, that can have subtly variable interpretations depending on context [7]. Here, replicability refers to the ability to obtain consistent results on repeated observations. Generalizability refers to the ability to apply a result from one sample population to a target population. For example, does a brain-based model of psychopathology generated from one sample predict psychopathology observed in a novel sample that may differ by one or many parameters (age, sex, location, socioeconomic status)? Results may be replicable within a held-out sample with similar sociodemographic characteristics but lack generalizability across one or more sociodemographic groups.

**Fig. 1:**
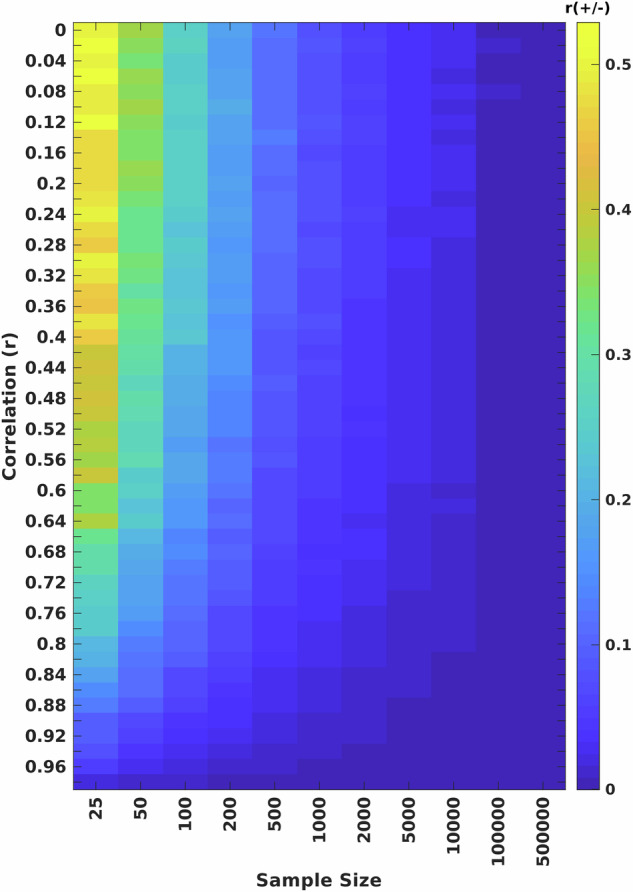
Sampling variability of correlations. Sampling variability (± of correlation on y-axis; magnitude of colored cells) of bivariate correlations (*r*) as a function of sample size (x-axis), ranging from *N*  = 25 to *N* = 500,000. Larger values represent higher sampling variability of a bivariate correlation across 1000 equivalently-sized subsamples. Sampling variability was quantified as the 99th percent confidence interval around an effect across 1000 bootstrapped samples.

The sample size of a study determines how replicable a result is likely to be given some true association strength (effect size) [8]. This is due to sampling variability, which refers to the variation in the observed effect estimate across random samples of a given sample size taken from a population [9]. Generally, as sample size increases, sampling variability decreases at a rate of √*n*. Fig. 1 provides an illustration from simulated data of the expected sampling variability of a correlation at a given sample size. As an example, the correlation between height and weight in the baseline Adolescent Brain Cognitive Development (ABCD) Study (*N* =  11,863, 9–10 years old) [10] is *r* = 0.63. However, if a researcher relies on a small sample (*N* = 25), previously common in many population psychiatric neuroimaging studies [6, 11], they can observe a correlation as strong as *r* = 0.95 or a correlation as weak as *r* = 0.25.

Bivariate association strengths between biological variables often exhibit associations closer to 0 than 1.0 [6, 12, 13]. In the case of a bivariate correlation of |*r*|  ≤ 0.10, common in population neuroscience [12, 14], there is high sampling variability around the estimate at samples in the tens to hundreds of individuals. For example, at an *N* = 25, the 99% confidence interval around an observed correlation of *r* = 0.10 is *r* ± 0.50. As confidence of an effect size increases substantially with large samples, increasing sample sizes into the thousands provides a straightforward solution to replication failures commonly observed in small sample population neuroscience [14–17].

Small sample sizes limit the ability to draw a confident conclusion about the relationship between two variables (e.g., brain connectivity and psychopathology symptoms). If inferential statistics are used to determine whether the association is significant, a small, underpowered sample will find either a statistically significant (*p* < 0.05), but inflated effect, a false negative (concluding no association exists when one does), or a false positive (concluding an association exists when one does not) [18, 19]. Because of publication bias [20, 21], the false positive or the inflated association will be published, but the false negative will not. Thus, small, underpowered sample sizes exhibit large sampling variability and all but guarantee erroneous published inference [13, 14]. Going forward, pre-registered reports may help address publication bias. In principle, meta-analytic approaches also can overcome single, underpowered small *N* studies by pooling across many small studies. However, overreliance on statistical thresholding for publication and data sharing perpetuates inflated associations in meta-analytic approaches [22]. Meta-analyses could be improved in an environment in which all data are shared without statistical thresholding. In either the framework of a consortium or meta-analysis of unthresholded data, the use of large samples can improve statistical power, and thus, replicability of small effects.

### Replicability improves with larger sample sizes

The replicability of an association depends on sample size, such that the replicability increases with increasing sample size [8]. This phenomenon can be illustrated using empirical data by quantifying the effect sizes of the largest brain-behavior associations (RSFC with fluid intelligence) across three consortia datasets (Human Connectome Project [*N* = 900], ABCD Study [*N* = 3928], and the UK Biobank [*N* = 32,725]).

**Fig. 2:**
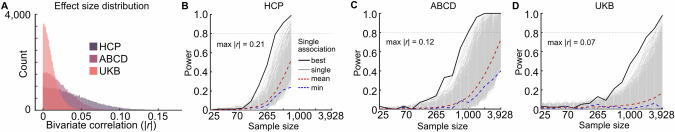
Effects of sample size on an exemplar brain-behavior association. **A** Effect size distribution (bivariate |r|) of resting-state functional connectivity edges with fluid intelligence for curated samples from the HCP (*N* = 900), ABCD (*N* = 3928) and UK Biobank (*N* = 32,572). Estimates of statistical power (y-axis) as a function of sample size (x-axis) for resting-state functional connectivity (RSFC) and fluid intelligence associations across all connections (brain features same across data sets), using (**B**) HCP (*N* = 900), (**C**) ABCD (*N* = 3928), and (**D**) UKB (*N* = 32,572), as reference datasets. Associations that passed statistical significance testing (*q* < 0.05, FDR corrected) in the respective full reference sample were included. In all panels, the black line (‘best’) represents the strongest association, the red dotted line (“mean”) represents the average statistical power across all significant (*q* < 0.05) RSFC with fluid intelligence associations, and the blue dotted line (“min”) represents the statistical power for the weakest suprathreshold association. Gray lines represent the statistical power for each statistically significant RSFC with fluid intelligence association. Each panel was scaled to the ABCD sample size for side-by-side comparison. Note the rightward shift for ABCD and UKB relative to HCP, demonstrating effects sizes are likely inflated even when using samplings in the hundreds (HCP) or under 10,000 (ABCD).

As the sample size of a dataset increases, the distribution of observable effect sizes shrinks (Fig. 2A). In the HCP sample, the largest effect size (RSFC with fluid intelligence) is *r* = 0.21 (Fig. 2B, black line). Although one may be tempted to conclude a univariate effect between the brain and fluid intelligence this large exists, evaluation of an even larger dataset shows that a correlation of this magnitude is a product of sampling variability. In the ABCD sample, the largest RSFC with fluid intelligence association is *r* = 0.12 (Fig. 2C, black line), a 50% reduction in maximum effect size compared to HCP. The minimum sample size required to be 80% powered at *P* < 0.05 (uncorrected) to detect the largest ABCD effect is *n* = 540 (the sample size at which the black line reaches 80% power in Fig. 2C). In the even larger UKB sample, the maximum association between RSFC with fluid intelligence shrinks further to *r* = 0.07 (UKB; Fig. 2D, black line), a two-thirds reduction from HCP. Thus, with UKB as the reference sample, 80% power to detect the largest RSFC with fluid intelligence association effect requires *N* = 1596 at *P* < 0.05 (*N* = 2376 at *P* < 0.05, FDR corrected for multiple comparisons). Notably, changing the statistical thresholding cannot improve the accuracy of a given effect size estimate relative to the true population effect. The use of large, well-powered samples can overcome sampling variability and improve replicability.

## Benchmarking current effect sizes

Effect sizes between brain connectivity and fluid intelligence are relatively small (*r*’s ~ 0.10). Only samples into the thousands are large enough to provide an accurate and precise estimate of these effects. Here, we benchmark associations between brain connectivity and mental health symptoms.

**Fig. 3:**
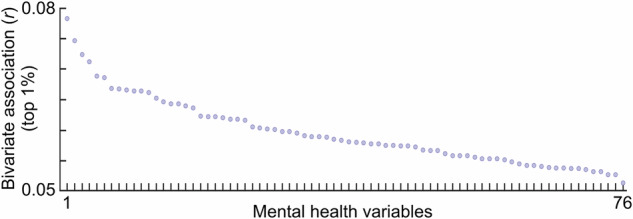
Mental health effect sizes. Univariate correlations (top 1%) between resting-state functional connectivity edges and an array of mental health variables. See Table S1 for the list of ordered variables.

Using the large ABCD Study as a reference dataset (*N* = 3928), mental health symptoms correlated with measures of brain structure and function maximally at *r* ~ 0.10 (Fig. 3, also see [12, 14]). This observation suggests that measures of mental health are even more weakly associated with brain measures than cognitive measures like IQ. Similarly, multivariate machine learning models used in brain-based prediction studies of mental health symptoms have exhibited relatively low prediction accuracies, requiring samples well into the thousands for replicability [23–25]. As such, only very large sample sizes (thousands) are powered to be effective for population-based psychiatric neuroimaging studies, whether within an association or predictive framework. Smaller samples will appear to have larger effect sizes and prediction accuracies but are likely to be inaccurate due to sampling variability and overfitting [15, 26].

## Increasing effect sizes in BWAS designs

Replicating small effects in population neuroscience (e.g., BWAS) designs necessarily requires large study samples (i.e., thousands of subjects or more). Concerned for the practical challenge this presents for study design, investigators have sought to identify measures and analysis strategies with larger effect sizes that may be identifiable with smaller samples [24, 25, 27]. We and others have promoted multivariate (vs. univariate) approaches as potentially allowing for less massive sample sizes to demonstrate replicable effects [14, 15, 23–25]. For example, multivariate strategies have demonstrated improved replicability within the study of cognitive variables, such as intelligence [14, 15]. However, it is important to note that reports of replication in samples of hundreds of individuals still relied on large samples for model training. Moreover, in contrast to cognitive variables, brain-based prediction of mental health phenotypes has not been replicated in similarly moderately-sized samples [14, 24, 25]. In any case, multivariate prediction accuracy also increases with increasing sample size [14, 15, 28]. Thus, as maximizing prediction accuracy is critical for eventual clinical translation [4, 16], we reaffirm the call for the largest samples possible for BWAS.

Alternative brain modalities have been used to try to identify stronger brain-behavior relationships [23]. For example, task-based fMRI has been reported to demonstrate larger univariate association strengths and multivariate prediction accuracies for some cognitive phenotypes than resting-state fMRI [24, 29, 30]. However, the interpretation of these increased effect sizes should be carefully considered. The introduction of a task induces a third variable problem. Specifically, task-specific behavior that is correlated to the target phenotype of interest obfuscates the independent contribution of brain activity or connectivity to target phenotype. For example, measurement of fMRI during a 2-back working memory task has been used to predict individual differences in various cognitive abilities [24, 29–31]. In this case, a cognitive task contrast is being used to predict an out-of-scanner cognitive measure (e.g., fluid intelligence). However, it has been shown that task-based prediction does not outperform resting-state once individual differences in performance are considered [14]. Thus, the degree to which a task produces larger brain-based prediction accuracies of a target phenotype may be dependent on how correlated the task performance is with the target phenotype (i.e., construct validity of the task relative to the target phenotype). For the same reason, we might expect brain-based prediction from a 2-back task to perform well for a cognitive variable but have limited additional unique explanatory power in prediction of psychopathology variables (e.g., internalizing/externalizing, anhedonia, etc.).

### A note on heterogeneity, validity, and measurement reliability

In addition to the need for larger samples, several other factors related to psychiatric diagnostics present challenges for psychiatric neuroimaging. Phenotypic heterogeneity, i.e., that multiple symptom patterns are allowable within diagnostic categories, and mechanistic convergence, whereby diverse individual or combined etiologies may manifest with similar functional outcomes, complicates one-to-one brain-based association and prediction of psychiatric pathology [32, 33]. Further, commonly used brain measures and/or behavioral assessments may often have poor construct validity with regard to psychiatric diagnosis (e.g., to what extent should RSFC be expected to reflect psychiatric symptoms?). These challenges have long been recognized and are themselves the primary motivation for establishing better biomarkers of psychiatric disease through imaging.

In addition to these caveats, one explanation for the low benchmark effect sizes and multivariate prediction accuracies seen in the larger datasets is that the brain measures and phenotypic assessments themselves are relatively unreliable, attenuating effect size estimates. Thus, there has been a push to improve the validity and reliability of brain connectivity and phenotypic measurement [16, 34, 35]. While we encourage this avenue of development, improved reliability alone is unlikely to improve effect sizes enough to support high replicability of small samples in population psychiatric neuroimaging.

## On generalizability in population psychiatric neuroimaging

Increased replicability of population-based psychiatric neuroimaging is an attainable goal with sample sizes into the thousands. However, while large samples are a prerequisite for generalizability, they are not necessarily sufficient to ensure generalizability of results to all populations [36–38]. A study sample must also accurately represent the target population of interest [39]. Brain-based studies of mental health may be particularly susceptible to this difficulty, as MRI scanners are largely located within urban and suburban centers of western, industrialized nations. Thus, neuroimaging studies have overrepresentation of white individuals, largely from higher socioeconomic strata (i.e., WEIRD samples) [40, 41]. For example, the samples used here totaling nearly 50,000 individuals included the HCP (USA, 76% white) [42], ABCD (USA, 73% white) and UK Biobank (United Kingdom, 95% white) [43, 44].

In addition to demographic representativeness, there are numerous reasons for poor generalizability of a machine learning model [45], including—but not limited to—overfitting, data leakage, research incentives, and shortcut learning. Shortcut learning refers to a phenomenon in which machine learning models learn a nontarget variable, rather than associations with the target variable of interest [46]. A concerning number of such events have transpired in the medical imaging community, including the use of machine learning to identify lungs with pneumonia [47] and Covid-19 [48]. In each instance, models did not learn the intended targets (disease), but rather background third variables (hospital codes and background luminosity, respectively).

The human brain is embedded within a complex environment, with innumerable variables that may covary with it and a target variable. This complexity is especially evident when considering individual differences in psychopathology (intended target) that often covary with symptom presentation related to demographics, socioeconomics, culture, and physiology, making generalizability of brain-based models an arduous task [28, 37, 49].

**Fig. 4:**
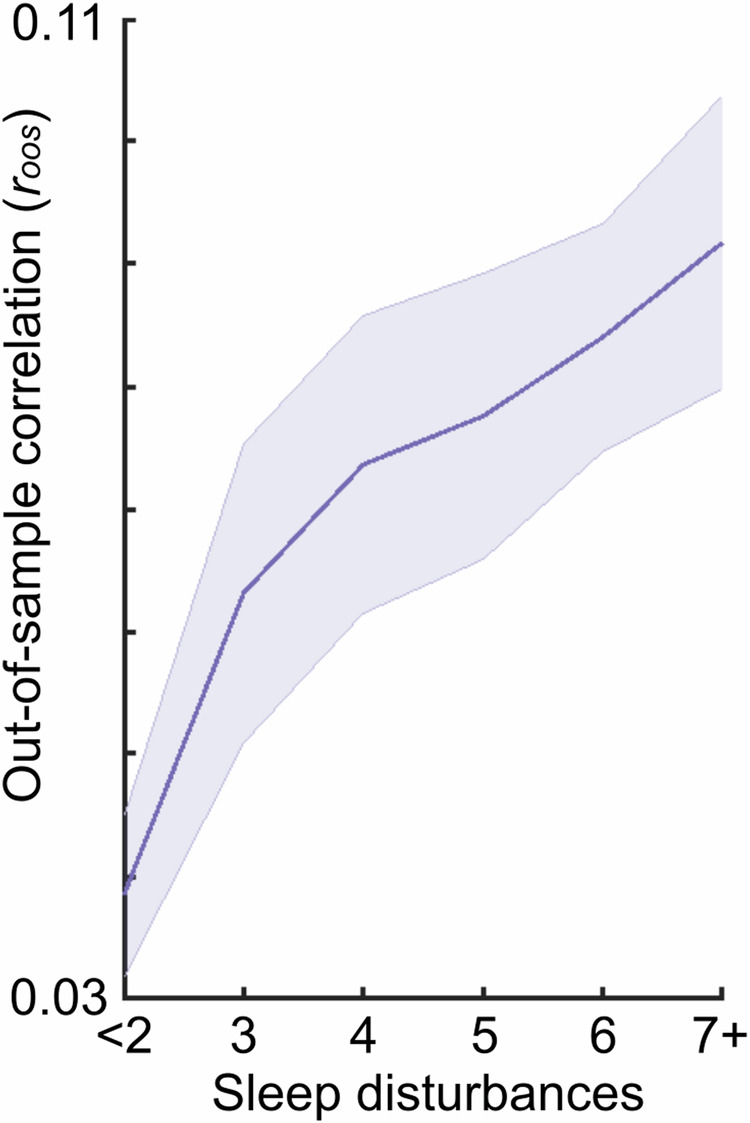
Generalizability of a brain-based model of mental health symptoms. Out-of-sample correlation (*r*_*oos*_; y-axis) of brain-based (RSFC) prediction of mental health symptoms (BIS-BAS) for varying samples drawn from the ABCD training dataset with varying levels of disturbances in initiating and maintaining sleep (i.e., sleep disturbances; x-axis). All training samples contained *N* = 400 individuals; the testing sample always contained *N* = 1964 individuals. Moving to the right on the x-axis indicates greater inclusion of individuals with sleep disturbances in the training sample. For example, “7+” includes individuals from the full range of sleep disturbances (from <2 to 7+). Increasing the range of sleep disturbances in the training sample improved the generalizability of brain-based models of mental health.

To illustrate the challenge of generalizing an observed brain-based association, we present an example of multivariate brain-based (RSFC) prediction of psychopathology symptoms (all items on the Behavioral Inhibition and Activation Systems (https://psycnet.apa.org/record/1995-00067-001). Specifically, we report the out-of-sample prediction using training sample subjects with different amounts of difficulty initiating and maintaining sleep (Fig. 4). Out-of-sample correlations (*r*_*oos*_) of brain-based models of psychopathology were low when the multivariate models were trained using only individuals without sleep disturbances. However, brain-based predictions of psychopathology symptoms increased when the training sample included individuals with sleep disturbances.

The dependency on the existence of sleep disturbances for superior prediction of psychopathology symptoms using brain data suggests that machine learning models may be learning associations between the brain and sleep, rather than the intended target of psychopathology symptoms. This demonstration provides a cautionary tale, such that even when a model appears to generalize, it may not be due to associations between the brain and the intended target (psychopathology), but rather an unmeasured third variable [46].

## Concluding remarks

Large consortia studies have revealed association strengths and prediction accuracies of population brain-based models of mental health symptoms to be relatively small. Use of the largest available samples for population psychiatric neuroimaging will improve replicability of association and prediction models [4, 28]. Historically, population psychiatric neuroimaging samples have lacked diversity. Larger samples in-and-of-themselves will not ensure models are generalizable to all populations. Studies with more diverse, representative, and equitable sampling and data aggregation efforts can improve generalizability and reduce scientific biases [41, 50].

The class of studies discussed in this perspective are specific to population approaches to psychiatric neuroimaging. This study design is distinct from cohort designs and within-person observational and intervention designs, including targeted treatment interventions [51, 52] and longitudinal deep phenotyping of brain connectivity [51]. For example, a study using a cohort design and repeated sampling of brain and depression symptoms within-person have revealed distinct signatures of trait- and state-level variability in depression [53]. Population, cohort and within-person approaches into the neural basis of mental health are complementary and will likely be mutually informative into the future. For in-depth discussion of cohort designs and within-person approaches applied to the translational goal of mechanistic inference [4, 54], we encourage the reader to Gell et al. [55] within this special issue.

The future for psychiatric neuroimaging has never been clearer and brighter. Large consortia efforts have clarified the sample size requirements for replicable and generalizable population approach to psychiatric neuroimaging. However, consortia are not the only path to larger, representative samples. Increased efforts around data aggregation and code sharing across single investigator-led studies will also promote improved replicability and generalizability. Moreover, publication and sharing of all data, regardless of statistical inference, will promote more accurate meta-analyses [54]. Altogether, population psychiatric neuroimaging studies will undoubtedly serve a critical purpose for informing smaller focused studies with the goal of mechanistic inference [4, 56] and eventual clinical application of functional MRI to psychiatry.

## Supplementary information

Methods

Mental health variable names (abcd_names)
