# Supplementary material for: Replicability and generalizability in population psychiatric neuroimaging
Source: Neuropsychopharmacology. 2024 Aug 30;50(1):52–7. doi: 10.1038/s41386-024-01960-w (PMC11526127; doi:10.1038/s41386-024-01960-w)
Supplement: Supplementary file 1 — Methods [file 41386_2024_1960_MOESM1_ESM.pdf]

Supplementary Methods

Datasets

Behavioral phenotype and neuroimaging (resting-state functional connectivity, cortical thickness) data were from the Human Connectome Project HCP [42] (HCP; 1200 Subjects Data Release), ABCD (3.0 release; Fast Track Release 3165 collection) datasets (*n* = 3,928; see ref [14] for details on participant information), and UK Biobank (UKB; *n* = 32,572 individuals from the January 2020 UKB release [57]). The ABCD Study obtained centralized institutional review board (IRB) approval from the University of California, San Diego. Each of the 21 sites also obtained local IRB approval. Ethical regulations were followed during data collection and analysis. Parents or caregivers provided written informed consent, and children gave written assent. All UKB and HCP participants also provided informed consent.

Imaging data

Details related to MRI acquisition and processing, see refs [42,57,58]. In each dataset (HCP, ABCD, and UKB), we extracted the timeseries from a total of 333 cortical ROIs [59], correlated and Fisher *z*-transformed them. The fluid intelligence measure was correlated (bivariate *r*) with each edge of the resting-state functional connectivity correlation matrix across participants.

Behavioral data

For HCP and ABCD, we used the age-adjusted fluid intelligence subscale from the NIH Toolbox. For the UKB, we used instance 2 of the fluid intelligence measure. Across all three datasets, behavioral phenotypes were used to correlate (bivariate *r*) with individual edges of resting-state functional connectivity (RSFC) data. Statistical power curves for each edge across the three datasets in Figure 2 followed bootstrapped resampling methods previously used by our group, detailed here [14].

Sampling variability simulation

For Figure 1, we quantified the sampling variability around a correlation, ranging from a correlation strength of $r = 0$ to $r = 0.98$ at varying sample sizes (N=25, 50, 100, 200, 500, 1,000, 2,000, 5,000, 10,000, 100,000, and 500,000). Two vectors of data, each with 10,000,000 elements (representing a population of participants) were created, centered around 0 with a standard deviation of 0.2. A correlation was induced between the two data vectors using Cholesky decomposition (chol.m). This represented the "ground truth" effect size in the data. Next, for each sample size, we subsampled the population vectors 1,000 times, with replacement, and calculated the correlation between the subsamples. Sampling variability was quantified as the 99% confidence interval around the correlation across subsamples at each sampling bin. This process was repeated for each correlation strength.

Generalizability of mental health symptoms

We tested the out-of-sample generalizability of multivariate models (canonical correlation analysis) of RSFC with mental health symptoms (all items on the BIS-BAS) for varying levels of disorders of initiating and maintaining sleep from the Sleep Disturbance Scale [60]. We used the predefined ABCD Discovery dataset as a training dataset and the ABCD Replication dataset as a test dataset [58]. We trained models using the ABCD data containing varying levels of representativeness with regards to sleep disturbances, ranging from low (containing fewer than two sleep disturbances) to high (over seven), with a total of 6 bins that were bootstrap resampled 500 times. Each ABCD training set consisted of $N = 400$ individuals for testing on the ABCD Replication dataset. The mean across the 500 resamples +/- 0.5 standard deviations is plotted in Figure 4.

References

57. Littlejohns TJ, Holliday J, Gibson LM, Garratt S, Oesingmann N, Alfaro-Almagro F, et al. The UK Biobank imaging enhancement of 100,000 participants: rationale, data collection, management and future directions. Nat Commun. 2020;11:2624.
58. Feczko E, Conan G, Marek S, Tervo-Clemmens B, Cordova M, Doyle O, et al. Adolescent Brain Cognitive Development (ABCD) Community MRI Collection and Utilities. bioRxiv. 2021:2021.07.09.451638.
59. Gordon EM, Laumann TO, Adeyemo B, Huckins JF, Kelley WM, Petersen SE. Generation and Evaluation of a Cortical Area Parcellation from Resting-State Correlations. Cereb Cortex. 2016;26:288–303.
60. Bruni O, Ottaviano S, Guidetti V, Romoli M, Innocenzi M, Cortesi F, et al. The Sleep Disturbance Scale for Children (SDSC). Construction and validation of an instrument to evaluate sleep disturbances in childhood and adolescence. J Sleep Res. 1996;5.
